# Observations on the Influence of Consanguinity as the Medium of Propagating Typhoid Fever

**Published:** 1875-09

**Authors:** T. S. Hopkins

**Affiliations:** Thomasville, Georgia


ATLANTA

MEDICAL AND SURGICAL JOURNAL.

VOL. XIII.] SEPTEMBER—1875. [No. 6.

### Original Communications.

### Observations on the Influence of Consanguinity as the Medium of Propagating Typhoid Fever.

BY T. S. HOPKINS, M.D., THOMASVILLE, GA.

I purpose in this report to present for consideration an unrecorded feature in the history of typhoid fever, the evidence of which is based upon many years of close personal observation. The existence of a feature so often witnessed and carefully noted, occurring almost invariably in the disease as coming under my observation for more than twenty-five years, is sufficiently evident to my mind to warrant me in viewing it as no less characteristic than the lesions of Peyer's glands.

The feature to which I desire to call attention is the propagation of typhoid fever through the ties of blood, which it often pursues with unrelenting tenacity and ferocity, to the almost entire exclusion of all beyond those ties. In the latter part of the year eighteen hundred and sixty, an article on this subject from me was published in the *Oglethorpe Medical Journal,* of Savannah, and the data upon which the proposition, up to that time, was based were given. Since then several opportunities have been afforded me for the collection of additional evidence. Upon the evidence I submit the case for consideration. In doing so I would respectfully ask, that though you "strike" you will "hear me;" and though you may be disposed to charge me with presumption or arrogance in daring to trespass upon the historical land-marks of a disease so long and ably defined by so many illustrious authorities, you will in future carefully inquire into the relationship of your typhoid patients, and note your observations on the subject presented. Upon such results I am willing to stake its existence. It is the duty of the physician to make known every discovery, whether made by accident or otherwise, that may tend to the amelioration of human suffering, or the prevention of disease. The utility of many valuable medicines has been discovered by accident, and though we daily witness their beneficial effects, we are often unable to explain their *modus operandi*. Their value, however, is in no way impaired or diminished by our ignorance of the manner in which they exert a controlling influence over disease. If I am correct in my view of the manner in which typhoid fever is propagated, my inability to solve the mystery and explain *why* it is so, would in no way detract from its value or lessen its utility. I have never been able in a single instance to discover a *local cause* for typhoid fever where it has occurred under my observation. It has usually appeared in our healthiest localities, where no cess pools, or leaking sewers, or animal or vegetable decomposition were present to be arraigned as factors. I have searched in vain for the supposed morbific agents considered necessary for its development, in every instance referred to in this report.

But it is not my intention to dwell upon the supposed cause or causes of the disease. I desire simply to show by the evidence to follow that when, by some occult and mysterious agency, of which we know nothing, the blood of man is poisoned, giving rise to a certain set of symptoms, with attendant special lesions, which, taken together, constitute typhoid fever, the same agent develops the same disease in those connected with him by the ties of blood, to the exclusion of others; and if the first case contracts the disease at a distance from home, and returns with it, he transmits or imparts this agency to his blood kin, developing the same disease, whilst all others, about him and equally exposed but not related to him, enjoy almost entire immunity. This power to impart the contagion to his blood kin, in preference to all others equally exposed, is shrouded in no less mystery than is the exciting cause of the disease. May we attempt an explanation of this by an appeal to idiosyncracies, those strange and inexplicable peculiarities so often met with in certain families, and doubtless connected with individual conformation and structure? We know that the members of certain families are so unpleasantly affected by the odor of certain fragrant flowers as almost do "die of a rose in aromatic pain." Other families suffer severe gastric derangement and urticaria from the least indulgence in shell fish; whilst others are affected most unpleasantly and sometimes alarmingly by the minutest portion of tartarized antimony. How common the remark, "the image of the father, or the mother"—the same eyes, and hair, and features; complexion, expression, propensities and peculiarities. If all these physical and mental family resemblances are inherited, why not the peculiar family predisposition, receptivity and susceptibility to the action of particular morbific agents? The diathesis is not communicable from man to man. It is a blood inherency, an expression of identity of conformation and structure in certain families; and when one member of a family so constituted is exposed to the exciting cause, typhoid fever is developed, and, regardless of the distance from home, on his return he propagates through this family predisposition, the disease from which he suffers, whilst others equally exposed, with very rare exceptions, escape. We are not without the knowledge of instances where individuals contracting typhoid fever away from home on their return communicate it to their immediate family, and they to other relatives who visit them, and these last, on their return, communicate it to their families, and through this communication, from relative to relative, the disease lurks in a community for months, and ceases only when it has exhausted the supply of consanguinity exposed to its influence; while with very few exceptions none disconnected by the ties of blood, however much exposed by contact, have had the disease. What stronger evidence could there be of the entire independence of typhoid fever, in such instances, of any local cause, or of the influence of consanguinity in its propagation? I do not wish to be understood in these remarks as conveying the idea that *all* connected by the ties of blood who may be brought in contact with the sick *must* have the disease. Such an opinion would be contrary to the history of the most virulent and fatal epidemics. I do mean, however, to be understood as asserting that from personal observation, when typhoid fever extends beyond the *first case* it strikes those connected by the ties of blood in preference to all others.

Liebermeister says a typhoid fever patient may be treated in a well ventilated ward without danger of the disease being contracted by nurses coming in contact with him, or by other patients. I have no doubt of the correctness of this assertion. It has been witnessed and proven in the well ventilated rooms of private residences in the country, when the patient was a *stranger in blood* to occupants of the house who were in contact with him. It would be a singular and rare circumstance indeed for a patient with typhoid fever to find in the wards of a hospital any one, either a nurse or patient, connected with him by the ties of blood, through which he might propagate the disease.

Flint, in his valuable work on the practice of physic, relates the case of a wealthy citizen who spent the summer in the country, leaving his house in the city in charge of two persons. In September he returned with his family and occupied the house. Very soon typhoid fever appeared in the family, five of whom had it. None escaped but the parents. A nephew who left the house after the disease appeared, and went into the country, also had it. He attributes the disease to a *local cause*, a defective waste pipe in the cellar, from which at times a bad odor had been perceived. Is it not strange, if the defective waste pipe was the cause, that the two persons in charge of the house, and who for the entire summer had been subjected to the bad odor, were not sick? The nephew went into the country, where he had the fever, but no one is said to have contracted it from him, nor did it extend beyond the immediate family, from the house in the city, to any one else. Not only was a peculiar family susceptibility to the exciting cause of typhoid fever evidenced in this instance, but very strong evidence in favor of the proposition that when developed it rarely extends beyond the ties of blood.

But it may be asked, what good will be derived from proving the influence of consanguinity in the propagation of typhoid fever? I answer, much. It would enable us to arrest its progress, by limiting and confining it to the first case. How? Why, by keeping away from the sick all persons connected with him by the ties of blood, and having him nursed entirely by those not so connected. So satisfied have I long been of the influence of this blood predisposition as a propagating medium, that I make it a rule always to advise that all relatives of the sick be kept away from him, otherwise they will in all probability contract the disease. I have never yet had cause to regret such advice.

With these remarks, I now present the evidence upon which they are based. If in the future you shall prove that the views advanced by me on this subject fail to stand the test of your observation, you will at least exhonerate me of having attempted to inaugurate a doctrine fraught with injury to the human race, and, as an act of professional charity, excuse me for having presented for your consideration the results of personal observation, with the hope of aiding in the arrest of a fearful malady and the mitigation of human suffering.

Instance 1.—In the month of May, 1855, typhoid fever prevailed on the "Dover Hall" plantation, Turtle River, Glynn county, Georgia. There were one hundred and twenty-five negroes on the place. The first case was that of Moses, an able bodied young man, twenty-four years of age. Soon after seeing Moses I was called to others, and my cases gradually increased to forty-eight. Having on a previous occasion, whilst treating typhoid fever on another plantation, noticed that it was confined to particular families about the yard, whilst the visitors from the surrounding plantations were not sick, I carefully inquired into the genealogy of each case occurring at "Dover Hall," and proved beyond a doubt that of the forty-eight, without an exception, every one was connected with Moses, the first case, by the ties of blood. The other negroes on the plantation were equally exposed by daily contact with the sick, yet not one but those connected to Moses had the disease. One of my last cases here I feared would prove a broken link in my chain of evidence. This was Nancy, aged about 35 years. She occupied isolated quarters at some distance from where the disease prevailed. I hunted up the evidence, however, and proved that she was the natural daughter of old George, the father of Moses.

No local cause whatever could be discovered for the occurrence or prevalence of the disease at this place. If there had been it is but resonable to suppose that the other negroes would have suffered equally with the relatives of Moses. "Dover Hall" is one of the most delightful and healthy places in the country. The quarters were comfortable, and the negroes better fed, better clothed, and less worked than any others in that section. Moses, a few days before he became sick, had returned from a visit to Brunswick.

Instance 2.—During my attendance at "Dover Hall," one of my cases, Renty, a young man, died suddenly with enormous tympanitis. Attributing the sudden supervention of tympanitis and speedy death to perforation of bowel from ulceration, I

disinterred the body, which contrary to my orders had been buried some twelve hours, and by careful examination established the correctness of my diagnosis. The examination was made at midday, in the month of June, the weather being very warm and the cadaver very offensive. For nearly a week the effluvium clung to me with a tenacity defying the most approved disinfectants. I could not, by any means used, divest myself of it.

It was but a short time after this autopsy that one of my sons, J. G. H., had typhoid fever; then another son, H. W. H.; then two daughters; then a first cousin of my children, on a visit to us. From the early part of June to the last day of October, I was not without a case of typhoid fever in my house. It is evident that in this instance the cause of the fever was infection, the materies morbi having been carried by me in my clothing, acting as fomites, from the post mortem to my house. J. G. was infected, the spark was lighted in the current of consanguinity, which it followed through five members of my family, passing by our nurses, our friends and our neighbors; there was not a case outside of my yard in the village. There was no discernable local cause. Waynesville, my residence, has always been considered one of the healthiest localities in the State.

I trust I shall be excused for relating here an incident occurring in the case of J. G. H. He was very low, helpless, and for several days speechless; his nourishment being poured into his mouth by the teaspoonful. While in this condition one of his brothers came into the room and informed me that there were some fine watermelons at the door for sale. He turned his head toward his brother and said to him, "Get me one if you have to steal it." The melon was immediately brought in, when he asked me to cut it in two and place a spoon in one half and lay it beside him on the bed, that he might "just look at it." I did as he requested, and just then was called into another room, where I remained some time. On my return, notwithstanding his promise not to eat, he was stuffing it in with a power far beyond any I imagined he possessed. He had eaten freely of it. From this time he began to improve, and was soon convalescent. Since then I rarely refuse my typhoid patients ripe watermelon.

Instance 3.—In the year eighteen hundred and sixty, Mr. J. R. R., of Waynesville, Georgia, purchased a family of negroes, consisting of the mother, a grown daughter and two sons, one a man and the other about fourteen years of age. On their arrival I was sent for to see the boy, whom I found ill of typhoid fever. I at once informed Mr. R. that in all probability the rest of the family would have the disease, but his other negroes, who nursed and occupied the same house with the sick, would escape. The boy was long sick, but recovered. His sister contracted the disease and died. The elder brother, who was a "runaway" at the time of purchase, hearing of the transfer, came in to his new master in perfect health, but very soon contracted the disease and died. No other case occurred. The locality is unsurpassed for healthfulness, and no local cause existed. These cases received every attention from Mr. R. and his family, who were much about them.

Instance 4.—In the year eighteen hundred and sixty-four, Mr. J. M., a member of the Georgia Cadets, in the service of the State, was sent home with typhoid fever. The residence of the family was in Glynn county. Mr. M. died. Immediately after his death, the family, consisting of father, mother, a lovely daughter aged about seventeen years, and two sons aged about twelve and fourteen years, moved into the piney woods, in one of the healthiest places in the whole county. Very soon the daughter contracted typhoid fever, with symptoms identical with those of the brother, and died. Then the father and then the next oldest son sickened of this same disease and died. No other case occurred at that locality nor anywhere in the neighborhood. They received every attention and the best of nursing from friends and neighbors, all of whom escaped. In this instance I was not the attending physician, but the facts related of them I vouch for. They were personal friends of mine, and I was posted with all that happened by the attending physician and the mother of the children.

Instance 5.—In the year eighteen hundred and seventy-two J. U., a citizen of Thomas county, residing near the Florida line, returned home from a railroad meeting at Savannah with fever, which soon proved to be typhoid. In this instance the father, mother and three children had the disease. There was no other case in the neighborhood. The disease lingered long in Mr. U.'s family; and as it is customary in the country for the neighbors to visit much the sick, many were exposed to the influence of the disease and escaped.

One of the ladies who assisted in nursing the sick, some three weeks after return home, suffered from a mild attack of typhoid fever. The length of time from immediate exposure to the occurrence of the fever would be rather against making this case an exception to the rule. This was a widow lady without family, and was the only case at her house. None of her friends or nurses who were constantly with her had the fever. In this instance Mrs. U., the wife, had the fever before any of the children. She was the cousin of her husband.

Instance 6.—In the year eighteen hundred and sixty-eight Mr. G. M., a citizen of Thomas county, residing six miles from Thomasville, on a plantation where typhoid fever had never before been known, sickened and died of the disease. Two of his daughters also died of the fever. After the death of the second daughter the mother, Mrs. M., had the fever. She was removed to Thomasville, to the house of her deceased husband's sister, where she continued sick for many weeks; during the time she was constantly attended and kindly nursed by the family, not one of whom had the fever. I will remark here that I have never known the wife take fever from the husband, or the husband from the wife, unless they were connected by the ties of blood. In this instance Mrs. M. did not have the disease until after the daughters had it. In this instance I was not the attending physician, though I visited the family, and the history given is from personal observation.

Instance 7.—Dr. F., a gentleman from Seymour Indiana, in the fall of 1873, being himself an invalid, removed with his family, consisting of himself, wife and daughter and three sons, to Thomas county, and located on a beautiful farm three miles from Thomasville. In the spring of 1874, typhoid fever appeared in his family, beginning with a son five years of age. From this case it spread, and the other two sons and daughter had it. The mother and father escaped. The disease continued for months in the family. I saw them in consultation with the doctor and father as often as my professional duties would permit. Those living near the doctor were frequently and daily at his house and around the bedside of his sick children. Notwithstanding this exposure by contact with the sick, and the effluvium from the excreta, no case occurred outside of the family. Frequently I would be asked by visitors if this disease was contagious. My reply was: "Not to you, unless you are connected by the ties of blood." This was an entirely new settlement; there was not an old house on the place; the water was excellent, and all the surroundings in perfect order.

